# Natural Selection as the Primary Driver of Codon Usage Bias in the Mitochondrial Genomes of Three *Medicago* Species

**DOI:** 10.3390/genes16060673

**Published:** 2025-05-30

**Authors:** Yingfang Shen, Leping Qi, Lijuan Yang, Xingxing Lu, Jiaqian Liu, Jiuli Wang

**Affiliations:** 1 College of Ecological Environment and Resources, Qinghai Minzu University, Xining 810007, China; syfnc@126.com (Y.S.); wang9li@outlook.com (L.Q.); liu000111222333444@163.com (J.L.); 2 State Key Laboratory of Tibetan Medicine Research and Development, Qinghai University, Xining 810016, China

**Keywords:** *Medicago*, mitochondrial genome, codon usage bias, natural selection, phylogenetic analysis

## Abstract

Objectives: Codon usage bias is a fundamental feature of gene expression that can influence evolutionary processes and genetic diversity. This study aimed to investigate the mitochondrial codon usage characteristics and their driving forces in three Medicago species: *Medicago polymorpha*, *Medicago sativa*, and *Medicago truncatula*. Methods: The complete mitochondrial genome sequences of the three species were downloaded from GenBank, and 21 shared coding sequences were screened. Codon usage patterns were analyzed using CodonW 1.4.2 and CUSP software. Key parameters, including the relative synonymous codon usage (RSCU), effective number of codons (ENC), codon adaptation index (CAI), codon bias index (CBI), and frequency of optimal codons (Fop), were calculated. Phylogenetic trees and RSCU clustering maps were constructed to explore evolutionary relationships. Results: The GC contents of the mitochondrial genomes followed the order of GC1 > GC2 > GC3. ENC values averaged above 35, while CAI, CBI, and Fop values ranged from 0.160 to 0.161, −0.078 to −0.076, and 0.362 to 0.363, respectively, indicating a weak preference for codons ending with A/U. Correlation and neutrality analyses suggested that codon usage bias was influenced by both mutation pressure and natural selection, with natural selection being the dominant factor. Fifteen optimal codons, predominantly ending with A/U, were identified. Phylogenetic analysis confirmed the close relationship among the three Medicago species, consistent with traditional taxonomy, whereas the RSCU clustering did not align with the phylogenetic relationships. Conclusions: This study provides insights into the mitochondrial codon usage patterns and their evolutionary determinants in Medicago species, highlighting the predominant role of natural selection in shaping codon usage bias. The findings offer a foundation for comparative genomic studies and evolutionary analyses and may be beneficial for improving genetic engineering and breeding programs of *Medicago* species.

## 1. Introduction

The genus *Medicago* (Fabaceae) comprises approximately 90 species globally, many of which are highly nutritious and ecologically valuable forage crops [1]. These plants are highly nutritious, with a high biomass and crude protein content, and rich in digestible nutrients and mineral elements, significantly reducing feed supplementation costs in animal husbandry [2]. Among these, *M. polymorpha* (MP) is used as a vegetable [3,4]. *M. sativa* (MS) is known for its salt tolerance and soil improvement capabilities [4,5]. *M. truncatula* (MT), closely related to *M. sativa*, is a model legume for genetic studies due to its small genome and nitrogen-fixing ability [6].

Mitochondria are essential organelles in eukaryotes, playing key roles in ATP synthesis and stress responses [7]. They are crucial for energy production and regulate various cellular processes, including Ca²⁺ homeostasis and apoptosis [8]. Plant mitochondrial genomes are also involved in stress response mechanisms and metabolic adjustments, making them vital for plant survival and adaptation [9]. As dynamic semi-autonomous organelles, mitochondria possess circular DNA that is capable of independent replication and transcription, and while maternal inheritance is common in plants, paternal inheritance has also been observed in certain *Medicago* species, such as *M*. *sativa*, indicating a complex inheritance pattern [10]. Recent advances in high-throughput mitochondrial genome sequencing have enabled comprehensive studies on the phylogeny and genetic diversity of forage legumes, including *M. polymorpha*, *M. sativa*, and *M. truncatula* [11,12].

As the primary carriers of precise genetic information, codons play a crucial role in biological inheritance and variation in nature [13]. As far as is currently known, in the universal genetic code, 22 amino acids are encoded by DNA, yet the majority of proteins are primarily composed of the 20 common amino acids [14]. There are 64 codons in total in living organisms (including 3 stop codons), which leads to the phenomenon that many amino acids are encoded by 1 to 6 different codons [15]. Different species or genomic regions exhibit significant variations in synonymous codon selection, a phenomenon termed codon usage bias (CUB) [14]. CUB is regulated by multiple factors, including mutation pressure, nucleotide composition, natural selection, transcription–translation efficiency, and environmental adaptation [14,16,17]. Through its regulation of translation efficiency/accuracy, co-translational protein folding, mRNA stability, and transcription levels, CUB effectively regulates the protein structure and gene expression, serving as a critical determinant of gene expression levels and cellular functions [18,19]. Therefore, investigating mitochondrial codon usage patterns in plants facilitates the identification of horizontal gene transfer events and phylogenetic relationships, enables the exploration of species evolution, and enhances our understanding of the molecular mechanisms underlying environmental adaptation [20]. While previous studies have characterized codon usage patterns in *Medicago* nuclear genomes and chloroplasts [20,21,22,23], no studies on mitochondrial codon usage bias in *Medicago* species have been reported.

Despite the significance of *Medicago* species in agriculture, the mitochondrial codon usage bias has remained unexplored. This study focuses on the mitochondrial genomes of three *Medicago* species, *M. polymorpha*, *M. sativa*, and *M. truncatula*. By analyzing the codon usage patterns and their driving forces in these mitochondrial genomes, our study not only fills this knowledge gap but also provides insights that could be beneficial for improving genetic engineering and breeding programs of *Medicago* species. This study may also enhance our understanding of the general principles governing codon usage bias in plant mitochondrial genomes, offering a foundation for comparative genomic studies and evolutionary analyses.

## 2. Materials and Methods

### 2.1. Acquisition of CDS from Mitochondrial Genomes of Three Medicago Species

The complete mitochondrial genome sequences of *M. polymorpha* (MP, accession number MW971562), *M. sativa* (MS, accession number ON782580), and *M. truncatula* (MT, accession number KT971339) were downloaded from the GenBank database of the National Center for Biotechnology Information (NCBI) (https://www.ncbi.nlm.nih.gov/ (accessed on 15 September 2024)). CDSs were screened based on the criteria of length >300 bp, start codon ATG, and stop codon TGA, TAA, or TAG. Redundant CDSs were filtered out by retaining only the longest CDS sequence. Ultimately, the mitochondrial CDSs that were common to all three species were retained for analysis [24]. This rigorous filtering process resulted in a final dataset of 21 high-quality, non-redundant CDSs that were common to all three species and suitable for our comparative analysis.

### 2.2. Codon Usage Parameter Analysis

The qualified coding sequences (CDSs) were consolidated into a FASTA file and analyzed using CodonW 1.4.2 and CUSP (https://bioinformatics.nl/cgi-bin/emboss/cusp, accessed on 12 October 2024) software. Key parameters included the following:

- Nucleotide frequencies at the third codon position: A3s, T3s, C3s, and G3s (frequency of adenine, thymine, cytosine, and guanine at the third synonymous codon position, respectively).
- GC content: GC3s (mean GC content at the third synonymous codon position), ATall/GCall (total AT/GC content), and GC1all/GC2all/GC3all (GC content at codon positions 1, 2, and 3).
- Codon bias indices: ENC (effective number of codons), CAI (codon adaptation index), CBI (codon bias index), RSCU (relative synonymous codon usage), and Fop (frequency of optimal codons).

For each CDS, GC1, GC2, and GC3 (GC content at codon positions 1, 2, and 3) were calculated. Pearson correlation analysis was performed to assess relationships among the codon count, GC1, GC2, GC3, GCall, GC3s, CAI, CBI, Fop, and ENC.

### 2.3. Neutrality Plot Analysis

Neutrality plots were constructed using GC3 (*x*-axis) and GC12 (the average GC1 and GC2; *y*-axis) values derived from the CDSs. Regression analysis (SPSS 20.0) was used to determine the slope of the GC3–GC12 relationship. A slope approaching 1.0 (diagonal alignment) indicates mutational pressure as the dominant factor shaping codon usage bias, while a slope near 0 suggests natural selection as the primary driver [24,25,26].

### 2.4. ENC Plot Analysis

The ENC values (*y*-axis) were plotted against GC3 (*x*-axis) with an expected ENC curve, calculated as follows:

ENC_expected_ = 2 + GC3 + 29/[GC3^2^ + (1 − GC3)^2^]

Genes clustering near the expected curve indicates mutational dominance, whereas deviations imply natural selection-driven codon bias [27].

### 2.5. PR2 Plot Analysis

A parity rule 2 (PR2) plot was generated with the following:

*x*-axis: G3/(G3 + C3)

*y*-axis: A3/(A3 + T3)

Central quadrant intersections (A=T, C=G) represent mutation-dominated codon usage. Vector distances from this point quantify directional deviations caused by selection–mutation interplay [28].

### 2.6. Identification of Optimal Codons

Genes were ranked by their ENC values, with the top and bottom 10% being selected to form high- and low-expression gene pools. Codons were classified as optimal if they met two criteria: RSCU > 1 (high-frequency) and ΔRSCU (RSCU-H–RSCU-L) ≥ 0.08 [29]. Here, RSCU-H represents the RSCU of the high-expression gene pool, while RSCU-L represents that of the low-expression gene pool.

### 2.7. Phylogenetic and RSCU Clustering Analysis

Mitochondrial CDSs from 10 species (8 Trifolieae taxa including *Medicago* species, plus the outgroups *Glycine max* and *Glycine soja*) were retrieved from NCBI (Table 1). Shared CDSs were aligned using MAFFT [30] (https://www.ebi.ac.uk/Tools/msa/mafft/ (accessed on 12 October 2024)), and a Maximum Likelihood (ML) phylogenetic tree was constructed in MEGA 11 [31] (1000 bootstrap replicates). RSCU-based hierarchical clustering was performed online in BioLadder [32] (https://www.bioladder.cn/ (accessed on 12 October 2024)) using Pearson correlation coefficients.

## 3. Results

### 3.1. Annotation and Analysis of Protein-Coding Genes

The mitochondrial genome sequences of *M. polymorpha*, *M. sativa*, and *M. truncatula* were 287,639 bp, 290,285 bp, and 271,618 bp in length, respectively. After screening, 21 shared CDSs (Table 2) were identified for subsequent analysis. These genes were functionally classified into groups, including ATP synthase, cytochrome c biogenesis, ubiquinol cytochrome c reductase, cytochrome c oxidase, maturases, NADH dehydrogenase, and ribosomal protein large/small subunits. These CDSs are often highly expressed and critical for mitochondrial energy production and stress responses. Their high expression levels may necessitate efficient translation, potentially influencing codon usage bias, as optimal codons are favored for efficient gene expression.

### 3.2. Analysis of Codon Usage Bias-Related Indices

**Figure 1:**
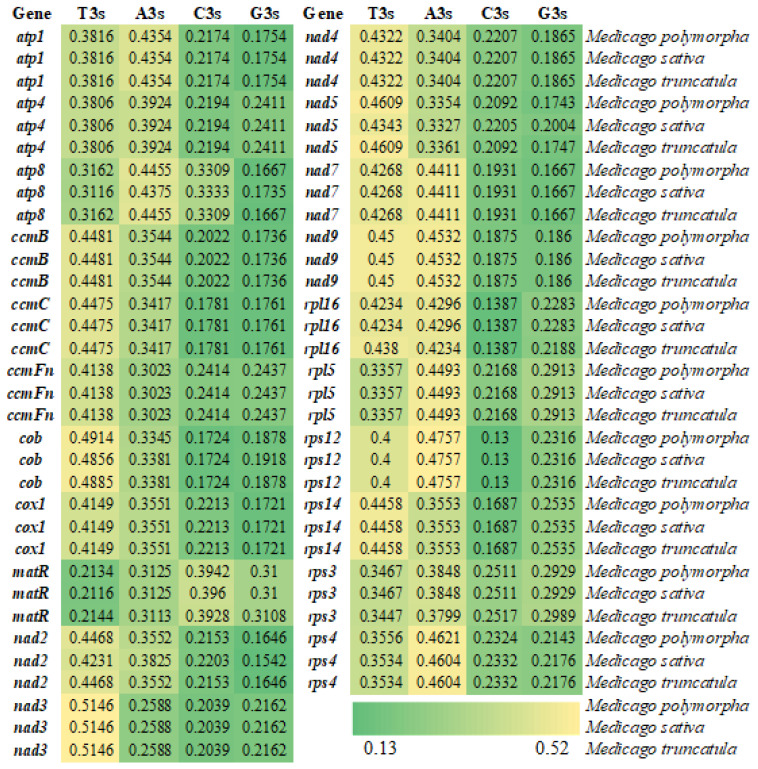
Heatmap of nucleotide composition at the third codon position in the mitochondrial genomes of three *Medicago* species.

**Figure 2:**
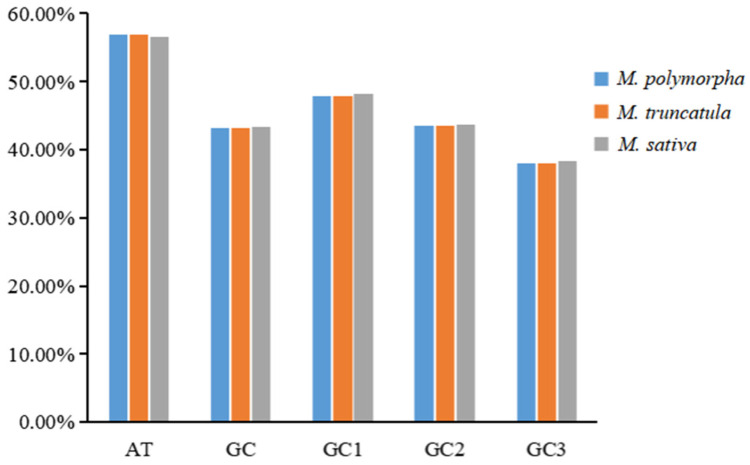
Base composition analysis of codons in the mitochondrial genomes of three *Medicago* species.

The nucleotide composition at the third codon position revealed a pronounced preference for A/U-ending codons across all three species, with the total AT contents ranging from 56.60% to 56.89% and GC contents ranging from 43.11% to 43.40% (Figure 1 and Figure 2). The GC contents at the first, second, and third codon positions (GC1, GC2, GC3) followed the trend of GC1 > GC2 > GC3, with all positions being below 50%. These results confirm a pronounced bias toward A/U bases in codon usage, particularly at the third position.

The effective number of codons (ENC) ranged from 47.11 to 61.00, with mean values of 54.41 (*M. polymorpha*), 54.47 (*M. sativa*), and 54.41 (*M. truncatula*). The codon adaptation index (CAI) averaged 0.160–0.161, significantly less than 1. The codon bias index (CBI) and frequency of optimal codons (Fop) showed weak bias, with mean values of −0.078 to −0.076 and 0.362–0.363, respectively (Table 3). These metrics collectively indicate low codon usage bias in the mitochondrial genomes of all three species.

**Figure 3:**
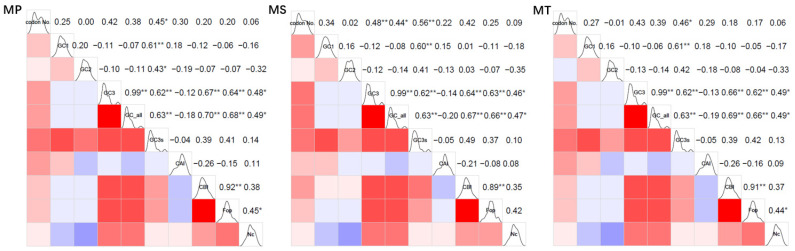
Correlation heatmap of codon usage indices in three *Medicago* species. codon No: codon number; GC1: GC content of codon base 1; GC2: GC content of codon base 2; GC3: GC content of codon base 3; GC_all_: total codon GC content; GC3s: GC content of mitochondrial genome synonymous codon base 3; CAI: codon adaptation index; CBI: codon preference index; Fop: frequency of optimal codon usage; ENC: effective codon count; **: significant correlation at the 0.01 level; *: significant correlation at the 0.05 level. In the upper-right section, numerical values quantify the correlation coefficients between the indices. The lower-left section uses colored blocks to visually depict these correlations, where red signifies a positive correlation and blue indicates a negative one. The intensity of the color reflects the strength of the correlation, with darker shades representing stronger associations.

The correlation analysis revealed significant relationships among various codon usage indices and GC content parameters (Figure 3). Specifically, GC3 and GCall were found to be strongly correlated across all species, with a correlation coefficient (r) of 0.99 and a *p*-value less than 0.01. Additionally, GC3s showed significant correlations with GCall (r = 0.63), GC3 (r = 0.62), and GC1 (r = 0.61). Furthermore, CBI and Fop were strongly linked to GCall (r ranging from 0.66 to 0.70) and GC3 (r ranging from 0.62 to 0.67). Finally, ENC demonstrated moderate correlations with Fop (r ranging from 0.44 to 0.49), GCall (r ranging from 0.47 to 0.49), and GC3 (r ranging from 0.46 to 0.49).

In summary, the mitochondrial GC3 content showed highly significant correlations with GCall in all three *Medicago* species (*p* < 0.01). Similarly, GC3s exhibited highly significant correlations with GCall, GC3, and GC1 (*p* < 0.01), indicating that the GC content strongly influences codon usage bias in their mitochondrial genomes. Furthermore, significant correlations were observed among the codon usage indices (CBI, Fop, ENC) and both GCall and GC3 (*p* < 0.05), demonstrating that nucleotide composition, particularly at the third codon position, plays a critical role in shaping codon usage preferences. These findings collectively highlight the substantial impact of base composition, especially at the third codon position, on codon usage patterns in the mitochondrial genomes of these species.

**Figure 4:**
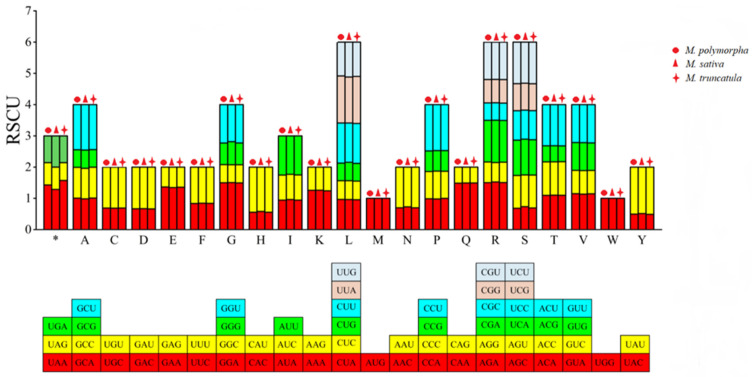
RSCU of codons in the CDSs of the mitochondrial genomes of three *Medicago* species. Each color in the stacked bars corresponds to a specific synonymous codon for the amino acids. The same colors are used in the RSCU bar chart above to represent the RSCU values of the respective codons. * indicates the stop codons.

The RSCU values of the codons in the mitochondrial genomes of the three *Medicago* species showed significant variation (Figure 4). For example, in *M. sativa*, the RSCU value of the arginine codon AGA was the highest (1.523), while the synonymous codon AGG had an RSCU value of only 0.625. Among the codons with RSCU > 1, 28 out of 30 in *M. polymorpha* and *M. truncatula* ended with A/U, while 27 out of 29 in *M. sativa* ended with A/U, indicating a preference for A/U-ending codons among the high-frequency codons in all three species.

### 3.3. Results of Neutrality Plot Analysis

**Figure 5:**
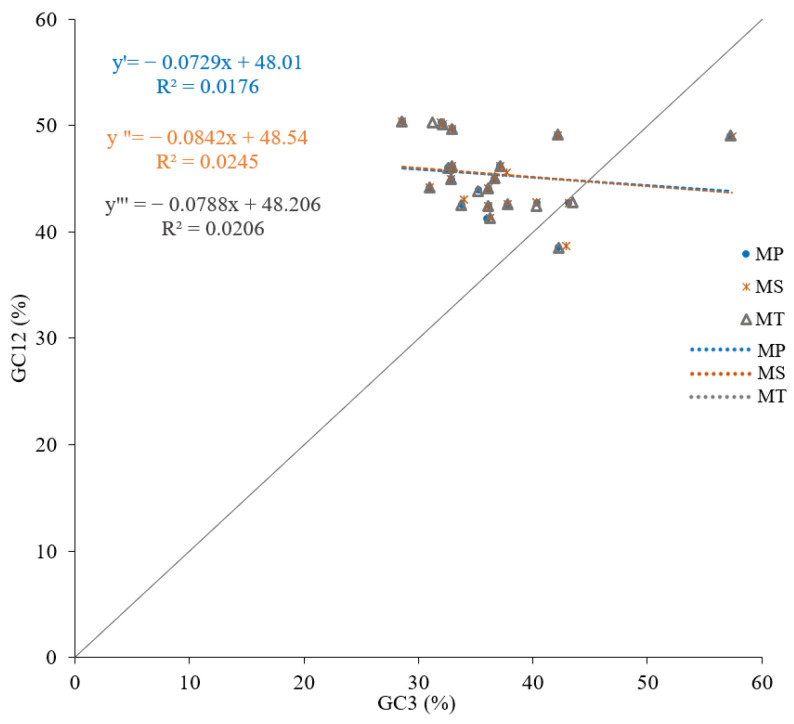
Neutrality plots of mitochondrial genomes in three *Medicago* species. Equations *y*′, *y*″, and *y*‴ represent *M. polymorpha*, *M. sativa*, and *M. truncatula*, respectively.

The neutrality plot analysis revealed that the GC3 content ranges were 28.57–57.25, 28.57–57.40, and 28.57–57.23 for *M. polymorpha*, *M. sativa*, and *M. truncatula*, respectively (mean values: 36.61, 36.80, and 36.60). The GC12 content ranges were 38.51–50.395 across all species (mean values: 45.34, 45.44, and 45.32). The regression coefficients between GC3 and GC12 were 0.1327 (*p* = 0.566), 0.1565 (*p* = 0.498), and 0.1435 (*p* = 0.535), with no significant correlation being observed (Figure 5). The weak correlation and low regression coefficients (−0.0842 to 0.0729) suggest that codon usage bias was shaped by multiple factors beyond just mutation pressure.

### 3.4. Results of ENC Plot Analysis

**Figure 6:**
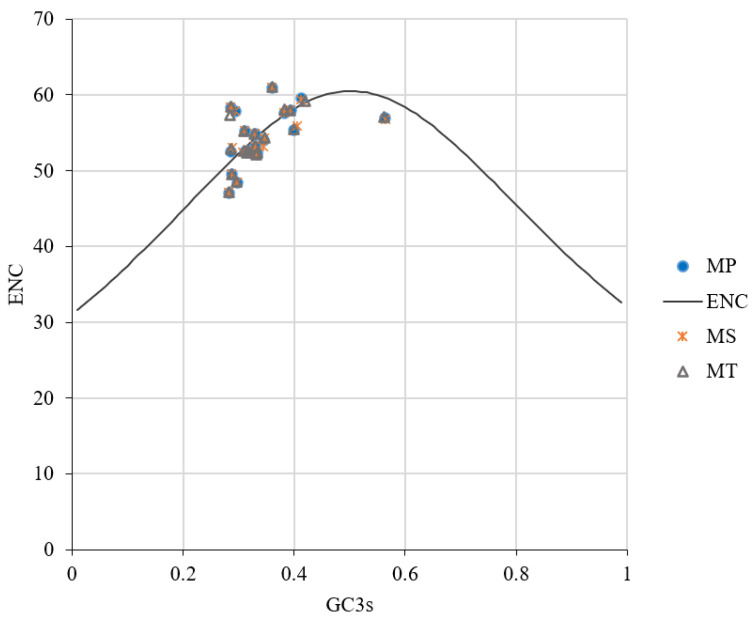
ENC plot analysis of mitochondrial genomes in three *Medicago* species.

The ENC plot analysis showed that most coding sequences deviated from the expected ENC curve (Figure 6), indicating that natural selection plays a dominant role in shaping codon usage preferences, with mutational pressure contributing minimally.

### 3.5. Results of PR2 Plot Analysis

**Figure 7:**
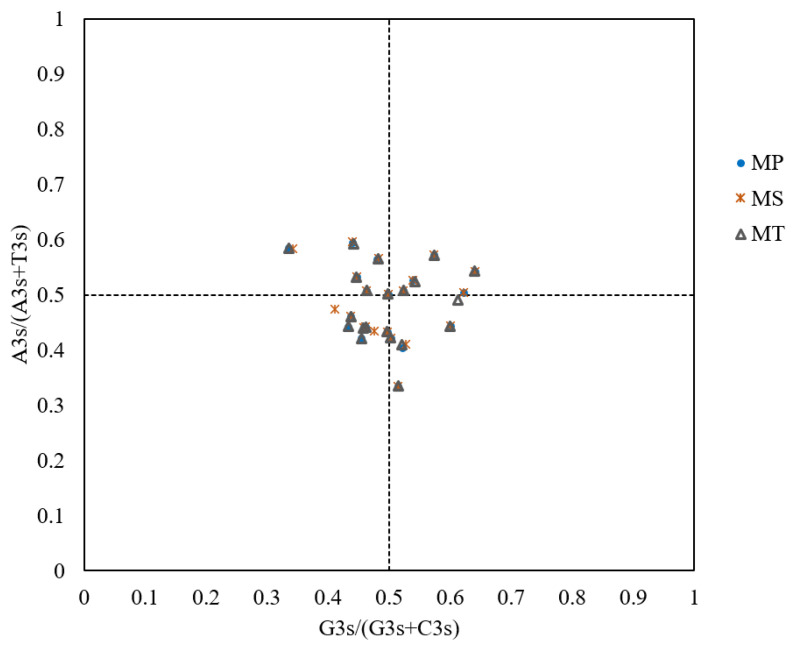
PR2 plot analysis of mitochondrial genomes in three *Medicago* species. The PR2 plot is divided into four quadrants by the central intersection point (G=C and A=T). Quadrant I (top-right) indicates an excess of A and G at the third codon position, while Quadrant III (bottom-left) indicates an excess of T and C. Quadrant II (top-left) indicates an excess of G and T, while Quadrant IV (bottom-right) indicates an excess of C and A.

The PR2 plot analysis revealed uneven distributions of genes across four quadrants (Figure 7). A bias toward T>A and C>G at the third codon position was observed, suggesting that codon usage bias arises from the combined effects of natural selection and mutational bias.

### 3.6. Optimal Codons

Optimal codons were defined as those with ΔRSCU ≥ 0.08 and RSCU > 1. *M. polymorpha*, *M. sativa*, and *M. truncatula* had 25, 25, and 26 high-expression codons, respectively. Among these, 16, 16, and 15 codons met both criteria and were identified as optimal codons (Table 4). Fifteen codons were shared across all three species, GCU (Ala), UGU (Cys), GAU (Asp), GAA (Glu), UUU (Phe), CAU (His), AAA (Lys), CUU (Leu), UUA (Leu), UUG (Leu), CAA (Gln), UCA (Ser), UCU (Ser), ACA (Thr), and UAU (Tyr), with fourteen ending in A/U and one in G.

### 3.7. Phylogenetic Relationships and RSCU Clustering Patterns

**Figure 8:**
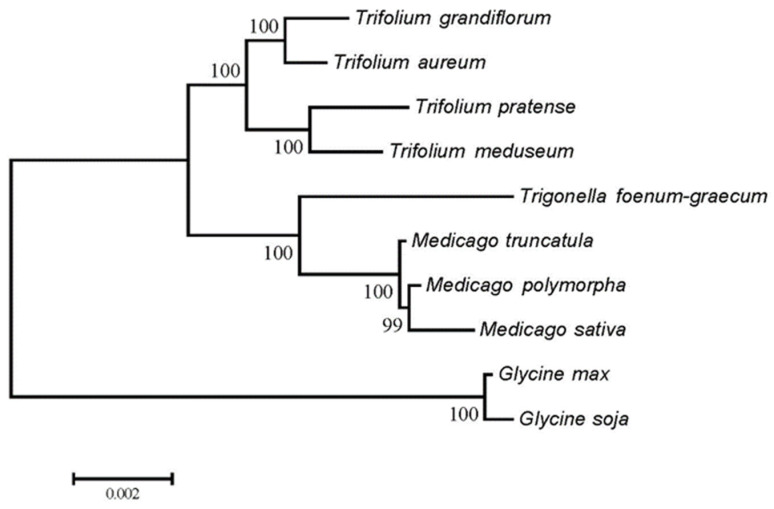
Phylogenetic tree of mitochondrial CDSs from 10 species.

**Figure 9:**
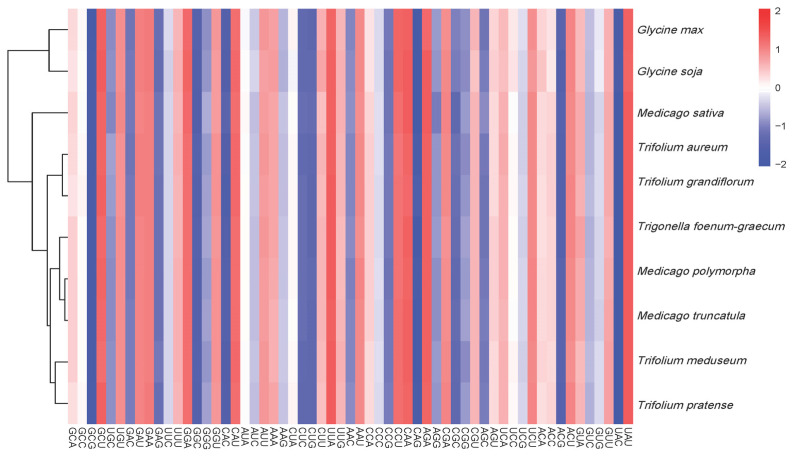
Dendrogram heatmap of RSCU-based clustering for mitochondrial CDSs from 10 species. The heatmap displays the RSCU values of the codons for each gene, with the color intensity reflecting the usage frequency of each codon.

The phylogenetic analysis based on mitochondrial CDSs (Figure 8) grouped the three *Medicago* species into a single clade (bootstrap support >99%), consistent with their close phylogenetic relationship. In contrast, RSCU-based clustering (Figure 9) placed *M. sativa* in a separate branch, while the remaining species formed mixed clusters, suggesting that RSCU patterns do not directly reflect evolutionary relationships.

## 4. Discussion

The analysis of 21 mitochondrial CDSs from the three *Medicago* species (*M. polymorpha*, *M. sativa*, and *M. truncatula*) revealed a GC content below 50% across all codon positions (GC1 > GC2 > GC3), with a pronounced bias toward A/U-ending codons. The pattern observed in the *Medicago* species aligns with findings from organelle genomes of other plants [13,33,34,35,36], indicating that codon usage bias likely enhances the translation efficiency and accuracy in both mitochondria and chloroplasts [37,38]. This suggests that the phenomenon is widespread across plant organelle genomes. The overlap of 15 shared optimal codons across the three species indicates conserved genetic traits, aligning with their close phylogenetic relationship. The discovery of optimal codons is valuable for enhancing the synthetic gene expression efficiency and stability in organelle genome engineering, such as in mitochondrial transformation and recombinant protein production [39,40].

The neutrality plot and ENC plot analyses indicated that natural selection predominates over mutation in shaping codon bias [36]. The PR2 plot analysis revealed a T>A and C>G bias at the third codon position, reflecting combined effects of mutation and selection [24,41]. The results of this series of analyses suggest that natural selection is the primary driver of codon usage bias in the mitochondrial genomes of *Medicago* species. However, it is important to consider the role of mutations and repair mechanisms in shaping these patterns. Recent studies on plant organellar DNA, particularly mitochondrial DNA, have shown that homologous recombination plays a crucial role in maintaining genome stability and influencing mutation rates [42]. For example, a reduced availability of homologous templates can accelerate nucleotide substitution rates in plant mitochondrial DNA due to an insufficient repair efficiency [43]. This highlights the importance of understanding the interplay between mutation rates and repair mechanisms in shaping mitochondrial genome evolution, which may also influence codon usage patterns in the mitochondrial genomes of *Medicago* species.

In the context of *Medicago* species, the observed codon usage patterns might be influenced by both natural selection and the underlying mutation rates. Mitochondria are essential for energy production and stress responses [44,45,46,47], and efficient translation of mitochondrial genes is vital for plant adaptation to environmental stresses [48,49]. The high usage of specific optimal codons could speed up the synthesis of respiratory chain proteins, which are crucial for maintaining mitochondrial function and plant survival under stress [37]. The observed codon usage patterns might also boost the mitochondrial transcript stability and protein synthesis efficiency, offering adaptive advantages in diverse conditions [50]. Therefore, future studies should explore the combined effects of natural selection and mutation rates on codon usage bias in plant mitochondrial genomes, particularly in *Medicago* species. This will provide a more comprehensive understanding of the evolutionary forces shaping mitochondrial genome evolution and their impact on plant adaptation and survival.

In this study, the topological discrepancy between RSCU-based clustering and a traditional phylogenetic tree based on mitochondrial gene sequences in *Medicago* and its close relatives may be attributed to several factors. First, RSCU clustering primarily reflects differences in codon usage preferences, which can be influenced by translational selection pressures, rather than solely evolutionary history [41,51]. Second, genetic drift and demographic events can cause random variations in codon usage, deviating from the phylogenetic signal [52]. Third, horizontal gene transfer can introduce genes with codon usage patterns typical of the donor organism, disrupting the native codon usage pattern [53,54]. Fourth, artificial selection may alter codon usage patterns, particularly in *M. sativa*, a key forage crop that has been subjected to prolonged selective breeding for enhanced agricultural traits, which could distinguish its codon usage from related species [55,56]. Finally, methodological differences between RSCU clustering and ML trees also contribute to discrepancies. RSCU clustering focuses on synonymous codon usage frequencies, while ML trees are based on sequence similarity and evolutionary models [57]. These differences in data types and analytical approaches can lead to varying topologies, providing complementary insights into the complex interplay between codon usage patterns and evolutionary relationships.

This study provides critical insights into the genetic diversity and codon usage evolution of *Medicago* species, offering a foundation for optimizing breeding strategies through the targeted manipulation of mitochondrial gene expression. Our findings highlight the potential for using codon optimization approaches to enhance the efficiency of genetic engineering in *Medicago* species. Furthermore, the identification of optimal codons in these species could aid in the design of synthetic genes for biotechnological applications. The observed patterns of codon usage bias and the role of natural selection in shaping these patterns contribute to our broader understanding of the evolutionary forces at play in plant mitochondrial genomes. This knowledge may be transferable to other plant species and could inform future research on mitochondrial gene expression optimization and its impact on plant growth and stress adaptation.

## 5. Conclusions

This study provides a comprehensive analysis of codon usage bias in the mitochondrial genomes of three *Medicago* species, revealing that natural selection is the primary driver shaping codon usage patterns. The identified 15 shared optimal codons, predominantly ending with A/U, are valuable for enhancing the synthetic gene expression efficiency in organelle genome engineering. The discrepancy between RSCU-based clustering and traditional phylogenetic trees underscores the complex interplay between codon usage patterns and evolutionary relationships. These findings not only fill a knowledge gap regarding *Medicago* mitochondrial codon usage bias but also offer insights into the general principles governing codon usage bias in plant mitochondrial genomes, potentially benefiting genetic engineering and breeding programs in *Medicago* species and advancing our understanding of evolutionary forces in plant mitochondrial genomes.

## Figures and Tables

**Table 1:** List of Genbank accession numbers for the 10 Fabaceae plants analyzed in this study.

Tribe	Genus	Species	Genbank Accession Number
Phaseoleae	*Glycine*	*Glycine max*	OR687435.1
Phaseoleae	*Glycine*	*Glycine soja*	MF955859.1
Trifolieae	*Medicago*	*Medicago polymorpha*	MW971562.1
Trifolieae	*Medicago*	*Medicago sativa*	ON782580.1
Trifolieae	*Medicago*	*Medicago truncatula*	KT971339.1
Trifolieae	*Trifolium*	*Trifolium grandiflorum*	MT039391.1
Trifolieae	*Trifolium*	*Trifolium aureum*	MT039392.1
Trifolieae	*Trifolium*	*Trifolium meduseum*	MT039390.1
Trifolieae	*Trifolium*	*Trifolium pratense*	MT039389.1
Trifolieae	*Trigonella*	*Trigonella foenum-graecum*	OP605625.1

**Table 2:** Functional classification of shared mitochondrial CDSs in three *Medicago* species.

Gene Group	Gene Names
ATP synthase	*atp*1, *atp*4, *atp*8
Cytochrome c biogenesis	*ccmB*, *ccmC*, *ccmFn*
Ubiquinol cytochrome c reductase	*cob*
Cytochrome c oxidase	*cox*1
Maturases	*matR*
NADH dehydrogenase	*nad*2, *nad*3, *nad*4, *nad*5, *nad*7, *nad*9
Ribosomal protein large subunit	*rpl*16, *rpl*5
Ribosomal protein small subunit	*rps*12, *rps*14, *rps*3, *rps*4

**Table 3:** CAI, CBI, Fop, and ENC values of mitochondrial genomes in three *Medicago* species.

Genes	*M. polymorpha*				*M. sativa*				*M. truncatula*			
	CAI	CBI	Fop	ENC	CAI	CBI	Fop	ENC	CAI	CBI	Fop	ENC
*atp1*	0.172	−0.082	0.362	52.73	0.172	−0.082	0.362	52.73	0.172	−0.082	0.362	52.73
*atp4*	0.154	−0.044	0.376	61	0.154	−0.044	0.376	61	0.154	−0.044	0.376	61
*atp8*	0.158	0.006	0.425	55.47	0.157	0	0.419	55.89	0.158	0.006	0.425	55.47
*ccmB*	0.158	−0.092	0.335	55.25	0.158	−0.092	0.335	55.25	0.158	−0.092	0.335	55.25
*ccmC*	0.145	−0.048	0.356	48.53	0.145	−0.048	0.356	48.53	0.145	−0.048	0.356	48.53
*ccmFn*	0.169	−0.026	0.391	57.95	0.169	−0.026	0.391	58.06	0.169	−0.026	0.391	57.95
*cob*	0.159	−0.157	0.3	52.58	0.161	−0.153	0.303	53.1	0.159	−0.157	0.3	52.87
*cox1*	0.176	−0.063	0.369	54.87	0.176	−0.063	0.369	54.87	0.176	−0.063	0.369	54.87
*matR*	0.146	−0.001	0.41	56.98	0.145	−0.001	0.41	56.72	0.146	−0.001	0.41	57.02
*nad2*	0.18	−0.119	0.342	52.59	0.188	−0.077	0.374	52.39	0.18	−0.119	0.342	52.59
*nad3*	0.18	−0.129	0.321	53.28	0.18	−0.129	0.321	53.28	0.18	−0.129	0.321	53.28
*nad4*	0.163	−0.071	0.353	52.15	0.163	−0.071	0.353	52.15	0.163	−0.071	0.353	52.15
*nad5*	0.17	−0.116	0.345	52.47	0.165	−0.109	0.347	53.16	0.17	−0.116	0.345	52.36
*nad7*	0.185	−0.102	0.359	49.55	0.185	−0.102	0.359	49.55	0.185	−0.102	0.359	49.55
*nad9*	0.168	−0.098	0.354	58.42	0.168	−0.098	0.354	58.42	0.168	−0.098	0.354	58.42
*rpl16*	0.16	−0.094	0.372	57.92	0.16	−0.094	0.372	57.92	0.16	−0.096	0.376	57.33
*rpl5*	0.167	−0.077	0.376	57.63	0.167	−0.077	0.376	57.63	0.167	−0.073	0.376	58.05
*rps12*	0.137	−0.098	0.355	47.11	0.137	−0.098	0.355	47.11	0.137	−0.098	0.355	47.11
*rps14*	0.133	−0.121	0.333	52.43	0.133	−0.121	0.333	52.43	0.133	−0.121	0.333	52.43
*rps3*	0.162	−0.056	0.387	59.61	0.161	−0.059	0.386	59.38	0.159	−0.069	0.379	59.24
*rps4*	0.127	−0.047	0.379	54.08	0.127	−0.047	0.379	54.33	0.127	−0.047	0.379	54.33

**Table 4:** RSCU values and optimal codons for mitochondrial genes in three *Medicago* species.

Amino Acid	*M. polymorpha*				*M. sativa*				*M. truncatula*			
	Codon	RSCU-H	RSCU-L	△RSCU	Codon	RSCU-H	RSCU-L	△RSCU	Codon	RSCU-H	RSCU-L	△RSCU
Ala	GCA	0.7619	1.0256	−0.2637	GCA	0.7619	1.0256	−0.2637	GCA	0.7619	1.0526	−0.2907
	GCC	0.1905	0.5128	−0.3223	GCC	0.1905	0.5128	−0.3223	GCC	0.1905	0.5263	−0.3358
	GCG	0.5714	0.9231	−0.3517	GCG	0.5714	0.9231	−0.3517	GCG	0.5714	0.9474	−0.376
	**GCU**	2.4762	1.5385	0.9377	**GCU**	2.4762	1.5385	0.9377	**GCU**	2.4762	1.4737	1.0025
Cys	UGC	0	0.4	−0.4	UGC	0	0.4	−0.4	UGC	0	0.4	−0.4
	**UGU**	2	1.6	0.4	**UGU**	2	1.6	0.4	**UGU**	2	1.6	0.4
Asp	GAC	0.2222	0.9231	−0.7009	GAC	0.2222	0.9231	−0.7009	GAC	0.2222	0.9231	−0.7009
	**GAU**	1.7778	1.0769	0.7009	**GAU**	1.7778	1.0769	0.7009	**GAU**	1.7778	1.0769	0.7009
Glu	**GAA**	2	1.4359	0.5641	**GAA**	2	1.4359	0.5641	**GAA**	2	1.4054	0.5946
	GAG	0	0.5641	−0.5641	GAG	0	0.5641	−0.5641	GAG	0	0.5946	−0.5946
Phe	UUC	0.4444	0.8182	−0.3738	UUC	0.4444	0.8182	−0.3738	UUC	0.4444	0.8182	−0.3738
	**UUU**	1.5556	1.1818	0.3738	**UUU**	1.5556	1.1818	0.3738	**UUU**	1.5556	1.1818	0.3738
Gly	**GGA**	1.5385	1.0667	0.4718	**GGA**	1.5385	1.0667	0.4718	GGA	1.5385	1	0.5385
	GGC	0.4615	0.8	−0.3385	GGC	0.4615	0.8	−0.3385	GGC	0.4615	0.8182	−0.3567
	GGG	0.4615	1.1556	−0.6941	GGG	0.4615	1.1556	−0.6941	GGG	0.4615	1.1818	−0.7203
	GGU	1.5385	0.9778	0.5607	GGU	1.5385	0.9778	0.5607	GGU	1.5385	1	0.5385
His	CAC	0.1818	0.4	−0.2182	CAC	0.1818	0.4	−0.2182	CAC	0.1818	0.4	−0.2182
	**CAU**	1.8182	1.6	0.2182	**CAU**	1.8182	1.6	0.2182	**CAU**	1.8182	1.6	0.2182
Ile	AUA	1.0645	1.1695	−0.105	AUA	1.0645	1.1695	−0.105	AUA	1.0645	1.1579	−0.0934
Ile	AUC	0.5806	0.8644	−0.2838	AUC	0.5806	0.8644	−0.2838	AUC	0.5806	0.8947	−0.3141
	AUU	1.3548	0.9661	0.3887	AUU	1.3548	0.9661	0.3887	AUU	1.3548	0.9474	0.4074
Lys	**AAA**	1.2222	1.0769	0.1616	**AAA**	1.2222	1.0606	0.1616	**AAA**	1.2222	1.0159	0.2063
	AAG	0.7778	0.9231	−0.1616	AAG	0.7778	0.9394	−0.1616	AAG	0.7778	0.9841	−0.2063
Leu	CUA	0.3913	0.6885	−0.2972	CUA	0.3913	0.6885	−0.2972	CUA	0.3913	0.6885	−0.2972
	CUC	0.3913	0.9836	−0.5923	CUC	0.3913	0.9836	−0.5923	CUC	0.3913	0.9836	−0.5923
	CUG	0.3913	0.5902	−0.1989	CUG	0.3913	0.5902	−0.1989	CUG	0.3913	0.5902	−0.1989
	**CUU**	1.4348	1.082	0.3528	**CUU**	1.4348	1.082	0.3528	**CUU**	1.4348	1.082	0.3528
	**UUA**	1.9565	1.4754	0.4811	**UUA**	1.9565	1.4754	0.4811	**UUA**	1.9565	1.4754	0.4811
	**UUG**	1.4348	1.1803	0.2545	**UUG**	1.4348	1.1803	0.2545	**UUG**	1.4348	1.1803	0.2545
Met	AUG	1	1	0	AUG	1	1	0	AUG	1	1	0
Asn	AAC	0.9091	0.7429	0.1662	AAC	0.9091	0.7429	0.1662	AAC	0.9091	0.7429	0.1662
	AAU	1.0909	1.2571	−0.1662	AAU	1.0909	1.2571	−0.1662	AAU	1.0909	1.2571	−0.1662
Pro	CCA	0.7143	1.2121	−0.4978	CCA	0.7143	1.2121	−0.4978	CCA	0.7143	1.2121	−0.4978
	CCC	0.8571	0.9697	−0.1126	CCC	0.8571	0.9697	−0.1126	CCC	0.8571	0.9697	−0.1126
	CCG	0.8571	1.0909	−0.2338	CCG	0.8571	1.0909	−0.2338	CCG	0.8571	1.0909	−0.2338
	CCU	1.5714	0.7273	0.8441	CCU	1.5714	0.7273	0.8441	CCU	1.5714	0.7273	0.8441
Gln	**CAA**	1.6364	1.2727	0.3031	**CAA**	1.6364	1.3333	0.3031	**CAA**	1.6364	1.3333	0.3031
	CAG	0.3636	0.7273	−0.3031	CAG	0.3636	0.6667	−0.3031	CAG	0.3636	0.6667	−0.3031
Arg	AGA	1.5429	1.7468	−0.2039	AGA	1.5429	1.7468	−0.2039	AGA	1.5429	1.7692	−0.2263
	AGG	0.1714	0.9873	−0.8159	AGG	0.1714	0.9873	−0.8159	AGG	0.1714	1	−0.8286
	CGA	1.2	0.9873	0.2127	CGA	1.2	0.9873	0.2127	CGA	1.2	1	0.2
	CGC	0.5143	0.6835	−0.1692	CGC	0.5143	0.6835	−0.1692	CGC	0.5143	0.6923	−0.178
	CGG	0.8571	0.6076	0.2495	CGG	0.8571	0.6076	0.2495	CGG	0.8571	0.6154	0.2417
Arg	CGU	1.7143	0.9873	0.727	CGU	1.7143	0.9873	0.727	CGU	1.7143	0.9231	0.7912
Ser	AGC	0.8571	0.5373	0.3198	AGC	0.8571	0.5373	0.3198	AGC	0.8571	0.5538	0.3033
	AGU	0.5143	1.0746	−0.5603	AGU	0.5143	1.0746	−0.5603	AGU	0.5143	1.0154	−0.5011
	**UCA**	1.3714	1.0746	0.2968	**UCA**	1.3714	1.0746	0.2968	**UCA**	1.3714	1.1077	0.2637
	UCC	0.5143	0.806	−0.2917	UCC	0.5143	0.806	−0.2917	UCC	0.5143	0.7385	−0.2242
	UCG	1.0286	0.9851	0.0435	UCG	1.0286	0.9851	0.0435	UCG	1.0286	1.0154	0.0132
	**UCU**	1.7143	1.5224	0.1919	**UCU**	1.7143	1.5224	0.1919	**UCU**	1.7143	1.5692	0.1451
Thr	**ACA**	1.5238	1.2414	0.2824	**ACA**	1.5238	1.2414	0.2824	**ACA**	1.5238	1.2857	0.2381
	ACC	1.1429	1.2414	−0.0985	ACC	1.1429	1.2414	−0.0985	ACC	1.1429	1.2857	−0.1428
	ACG	0.7619	0.6897	0.0722	ACG	0.7619	0.6897	0.0722	ACG	0.7619	0.5714	0.1905
	ACU	0.5714	0.8276	−0.2562	ACU	0.5714	0.8276	−0.2562	ACU	0.5714	0.8571	−0.2857
Val	GUA	1.1111	1.2683	−0.1572	GUA	1.1111	1.2683	−0.1572	GUA	1.1111	1.2683	−0.1572
	GUC	0.8889	0.878	0.0109	GUC	0.8889	0.878	0.0109	GUC	0.8889	0.878	0.0109
	GUG	0.4444	0.9756	−0.5312	GUG	0.4444	0.9756	−0.5312	GUG	0.4444	0.9756	−0.5312
	GUU	1.5556	0.878	0.6776	GUU	1.5556	0.878	0.6776	GUU	1.5556	0.878	0.6776
Trp	UGG	1	1	0	UGG	1	1	0	UGG	1	1	0
Tyr	UAC	0.3333	0.7273	−0.394	UAC	0.3333	0.7273	−0.394	UAC	0.3333	0.6667	−0.3334
	**UAU**	1.6667	1.2727	0.394	**UAU**	1.6667	1.2727	0.394	**UAU**	1.6667	1.3333	0.3334

High-frequency codons (RSCU > 1) are underlined; bolded codons indicate optimal codons. SCU-H represents the RSCU of the high-expression gene pool, while RSCU-L represents that of the low-expression gene pool.

## Data Availability

The datasets used and/or analyzed during the current study can be obtained from the corresponding authors upon reasonable request. The datasets analyzed during the current study are available on the following website: https://www.scidb.cn/s/qYVzUv (accessed on 8 April 2025).

## Abbreviations

The following abbreviations are used in this manuscript:

CAI	Codon adaptation index
CBI	Codon bias index
CDS	Coding sequences
CUB	Codon usage bias
ENC	Effective number of codons
GRAVY	Grand Average of Hydropathicity
ML	Maximum likelihood
RSCU	Relative synonymous codon usage
SCUB	Synonymous codon usage bias
MP	*Medicago polymorpha*
MS	*Medicago sativa*
MT	*Medicago truncatula*
GC1	GC content at codon positions 1
GC2	GC content at codon positions 2
GC3	GC content at codon positions 3
GC12	Average GC1 and GC2
GCall	Total GC content
